# Raiders of the Lost Correlation: A Guide on Using Pearson and Spearman Coefficients to Detect Hidden Correlations in Medical Sciences

**DOI:** 10.7759/cureus.11794

**Published:** 2020-11-30

**Authors:** Alessandro Rovetta

**Affiliations:** 1 Mathematical, Statistical and Epidemiological Models, Technological and Scientific Research, Redeev Srl, Naples, ITA; 2 Mathematical, Statistical and Epidemiological Models, Research and Disclosure Division, Mensana Srls, Brescia, ITA

**Keywords:** correlation, statistics, medical physics, medical statistics, pearson, spearman

## Abstract

Pearson and Spearman correlations are important tools for all scientific fields and are widely used in medical sciences, especially during the current COVID-19 pandemic emergency. This technical report has shown that conventional criteria for evaluating the adoption of these coefficients can conceal substantial scientific information regarding correlations that occur above or below a certain threshold. In particular, the Pearson coefficient can reveal hidden correlations even when data are not normally distributed. Finally, an optimized operational guide to reveal any hidden correlation is reported.

## Introduction

The search for statistical correlations between two data distributions constitutes one of the fundamental elements of scientific research [1-4]. Particularly in the fields of public health, social sciences, infoveillance, and epidemiology, these can provide important information on risk perception and the spread of viruses and bacteria [5-8]. The two most frequently used correlation indices are those of Pearson and Spearman: the first one measures the linear relationship between two continuous random variables and is adopted when the data follows a normal distribution while the second one measures any monotonic relationship between two continuous random variables and is adopted when the data do not follow a normal distribution; both range from -1 to 1 [1-4]. A correlation (ρ) is often defined in medicine as very strong (|ρ| > 0.7), moderate (0.7 ≤ |ρ| < 0.5), fair (0.5 ≤ |ρ| ≤ 0.3), or poor (|ρ| < 0.3) [3]. Nonetheless, it is customary to evaluate its significance based not only on the ρ value itself but also on the relative p-value [3]. The first problem in this approach is precisely the meaning of the p-value: some authors believe that exceeding the significance threshold α implies the immediate acceptance of the null hypothesis [9]; others assert that the p-value should be used as an index of the evidences found against the null hypothesis [10-12], and others conclude that the p-value in itself does not provide any information on the validity of the model used [13-14]. However, all cited authors agree that the mere violation of the significance threshold is not a criterion for the rejection of a statistical relationship. Therefore, in this paper, no significance threshold has been fixed and p-values ​(p) ​and Pearson (R) and Spearman (r) coefficients were used to evaluate the statistical significance and the strength of the correlations analyzed.

## Technical report

Is it okay to avoid the Pearson coefficient when the data are not normally distributed?

There are various methods to evaluate whether a data series is normally distributed: some finer, such as the Shapiro-Wilk test, others coarser, such as the standard errors-test for kurtosis and skewness [15-16]. In this paper, the latter has been used together with the graphical representation of every distribution. In Table 1, despite data not being normally distributed in most cases, we can see how the Pearson coefficient is able to highlight monotonous trends. Clearly, in the proposed situation, the Spearman coefficient is more appropriate since it perfectly detects this relationship. On the other hand, this shows that it is wrong to state that Pearson's coefficient is only useful when data are normally distributed although it remains true that it would not be able to identify certain non-linear correlations.

**Table 1:** Comparison between Pearson and Spearman correlations on data distributions printed through monotone polynomial functions K-test = Kurtosis test, S-test = Skewness test, R = Pearson’s correlation value, r = Spearman’s correlation value, D% = Percentage difference between R and r

	x	x^2	x^3	x^4	x^5	x^6	x^7	x^8	x^9	x^10
	1	1	1	1	1	1	1	1	1	1
	2	4	8	16	32	64	128	256	512	1024
	3	9	27	81	243	729	2187	6561	19683	59049
	4	16	64	256	1024	4096	16384	65536	2.62E+05	1.05E+06
	5	25	125	625	3125	15625	78125	3.91E+05	1.95E+06	9.77E+06
	6	36	216	1296	7776	46656	2.80E+05	1.68E+06	1.01E+07	6.05E+07
	7	49	343	2401	16807	1.18E+05	8.24E+05	5.76E+06	4.04E+07	2.82E+08
	8	64	512	4096	32768	2.62E+05	2.10E+06	1.68E+07	1.34E+08	1.07E+09
	9	81	729	6561	59049	5.31E+05	4.78E+06	4.30E+07	3.87E+08	3.49E+09
	10	100	1000	10000	1.00E+05	1.00E+06	1.00E+07	1.00E+08	1.00E+09	1.00E+10
K-test	-0.77	-0.48	0.20	0.96	1.71	2.40	3.02	3.57	4.05	4.46
S-test	0	0.87	1.47	1.92	2.28	2.58	2.82	3.03	3.21	3.35
R	1	0.97	0.93	0.88	0.84	0.80	0.77	0.74	0.72	0.70
r	1	1	1	1	1	1	1	1	1	1
Δ%	0	2.61	7.71	13.42	19.13	24.64	29.88	34.80	39.42	43.73

**Figure 1:**
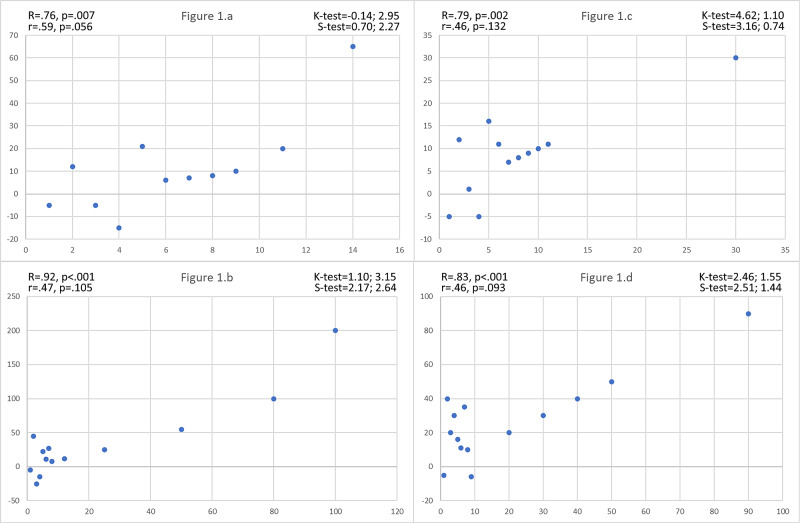
Comparison between the Pearson and Spearman coefficients in data distributions that show correlations beyond a specific threshold R = Pearson’s correlation value; r = Spearman’s correlation value, K-test = Kurtosis test, S-test = Skewness test

However, there is a more relevant aspect to discuss. in Figure 1, we can observe a peculiar statistical phenomenon, that is, a sequence of monotonic correlations that occur only when a certain threshold is exceeded. In this specific case, although the data is not normally distributed, Pearson's coefficient is even more effective than Spearman's, for it gives more weight to higher values (Figure 1).

In these examples, the hidden correlations are visible to the naked eye. But, when dealing with hundreds of distributions, it is not always possible to graph each data series. Thus, this method can be effective and efficient in revealing such hidden phenomena. When this happens (i.e. when Pearson's R is larger and more significant than Spearman's r), it is important to interpret it as a signal of plausible correlations.

This method works with all monotonic correlations, provided that all correlated values are greater than those preceding the threshold. Similarly, the same rules also apply to correlations that occur below certain thresholds.

How to discover correlations hidden in large data variability?

**Figure 2:**
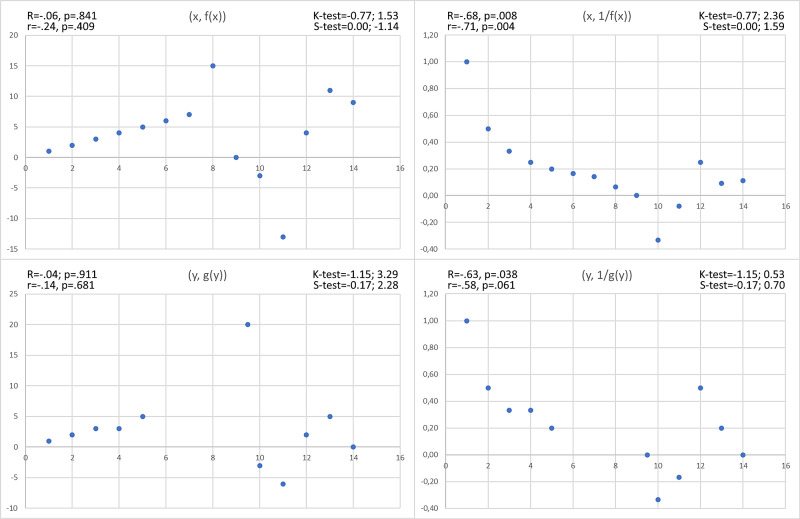
Hidden correlations revealed by the Pearson and Spearman coefficients through the reciprocal 1/k of the values k K-test = Kurtosis test, S-test = Skewness test, R = Pearson’s correlation value, r = Spearman’s correlation value

It remains questionable how to behave when the correlated values are lower than the unrelated values. A quick but rough method is to calculate, for each hypothetically dependent value *k ≠ 0*, the quantities *1/k* ​​and redo the operation. Although the nature of the correlation is distorted, in doing so, it is possible to signal the presence of a local monotonic relationship between the two variables (Figure 2).

**Figure 3:**
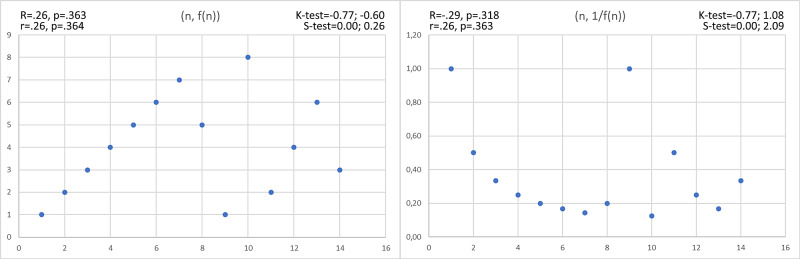
Hidden correlations not revealed by the Pearson and Spearman coefficients K-test = Kurtosis test, S-test = Skewness test, R = Pearson’s correlation value, r = Spearman’s correlation value

Nevertheless, this method is ineffective when correlated values ​​have the same magnitude as unrelated values (Figure 3).

Thus, when even the latter procedure cannot identify any hidden correlations, finer methods could be used. For example, these could be revealed via cyclic iterations: supposing we have two data distributions that vary from 1 to n, we first search for the correlation between all the pairs; then, we redo the calculation between *1* and *n-1*, between *1* and *n-2*, and so on. After that, the whole operation must be repeated starting from pair *2* (ergo, we search for the correlation between *2* and *n*, then between *2* and *n-1*, and so on).

A real example

In the early stages of the COVID-19 epidemic, the web interest of Italian netizens in the novel coronavirus was correlated with the number of cases per region above a certain threshold as shown in Table 2.

**Table 2:** Italian netizens' web interest in COVID-19 during the early stages of the pandemic in Italy (from February 20 to February 25, 2020): correlations between COVID-19 cases and web interest and between the number of medical swabs and web interest RSV = Relative search volume

	Region	Coronavirus RSV	COVID-19 Total Cases	Medical Swabs
1	Abruzzo	61	0	5
2	Basilicata	65	0	0
3	Calabria	63	0	2
4	Campania	67	0	10
5	Emilia-Romagna	84	26	391
6	Friuli-Venezia Giulia	66	0	89
7	Lazio	60	3	124
8	Liguria	68	1	39
9	Lombardy	100	240	3700
10	Marche	71	0	21
11	Molise	57	0	0
12	PA Trentino-Alto Adige/South Tyrol	60	1	4
13	Piedmont	82	3	141
14	Puglia	60	0	0
15	Sardinia	45	0	1
16	Sicily	54	3	5
17	Tuscany	69	2	296
18	Umbria	73	0	8
19	Valle d'Aosta	77	0	7
20	Veneto	79	43	3780
	Kurtosis-test	1.25	16.58	6.27
	Skewness-test	1.35	7.67	5.19
	Pearson R (p-value)		.68 (.001)	.63 (.003)
	Spearman r (p-value)		.41 (.07)	.75 (.0002)

In this case, despite the data not being normally distributed, the use of Spearman's correlation alone would not have highlighted the first correlation, which is most likely of a causal nature. The data were collected using the Google Trends tool and the website of the Italian Civil Protection Department (URL: https://gisanddata.maps.arcgis.com/apps/opsdashboard/index.html#/b0c68bce2cce478eaac82fe38d4138b1).

## Discussion

The aim of this technical report is to provide a guide for the appropriate use of the Pearson and Spearman correlation coefficients, showing that the data (non-)normality should not be the sole criterion for their adoption or rejection. Indeed, phenomena capable of manifesting and correlating above a certain threshold are known in the literature [7,17-19]. This paper shows that Pearson’s coefficient can reveal such hidden phenomena even when statistical tests suggest that data groups are not normal. Furthermore, the simultaneous use of both correlations allows to compensate for some potential failures of normality tests. In fact, the kurtosis and skewness standard errors-test is efficient but can be inaccurate while the Shapiro-Wilk test is more reliable but also operationally complex [15]. Therefore, if we have a pronounced Pearson correlation and a weak Spearman correlation, there may be a “correlation threshold”, i.e. we need to investigate further. If we have a weak Pearson correlation and a pronounced Spearman correlation, the relationship is likely to exist, but we must make sure that the data is not normally distributed; otherwise, further investigation is required. If both Pearson and Spearman correlations are pronounced, the correlation holds. Finally, if both correlations are weak, we need to recalculate them using the reciprocal of the hypothetically dependent variable to unmask any other possible hidden correlation. Alongside that, the only truly comprehensive and complete method for detecting hidden correlations is the cyclical search for correlations between data subsets: in fact, all the methods listed above can fail in their purpose when dealing with data distributions that contain hidden correlations between values similar ​​to those non-correlated. However, these procedures can drastically skim the data on which it is necessary to act via the cyclic-iterative method.

Some plausible scenarios in which it is legitimate to expect hidden causal correlations are: i) the effects of air pollution, where exceeding specific thresholds can cause an increase in population mortality and disease due to an impairment of the immune and respiratory systems [5,18], ii) the levels of interest, stress, and anxiety among the population, which can reach high values ​​when negative news exceeds a particular number [7], iii) in the specific case of the novel coronavirus, exceeding a certain value of the population density could have an important role in increasing the spread of severe acute respiratory syndrome coronavirus 2 (SARS-CoV-2) [6,18,20], and iv) particulate matter could act as a viruses-carrier, especially beyond specific thresholds [17-18].

## Conclusions

When data distributions are numerous, it is always recommended to calculate both the Pearson and Spearman correlations. To highlight hidden correlations on continuous data (X, Y), it is also important to recalculate both correlations on data (X, 1/Y). When even this procedure is not able to detect any hidden correlation and there are valid reasons to support its existence, it is necessary to resort to cyclic-iterative methods.
